# Incidentally found parotid gland lesion in ^18^F-FDG PET/CT for staging evaluation of patients with hepatocellular carcinoma: remote possibility of metastatic tumor or second primary salivary gland malignancy

**DOI:** 10.1186/s12893-024-02422-2

**Published:** 2024-04-24

**Authors:** Jin Hyung Jung, Yoon Se Lee, Young Ho Jung, Seung-Ho Choi, Soon Yuhl Nam, Hyo Jung Cho, Minsu Kwon

**Affiliations:** 1 Department of Otorhinolaryngology-Head and Neck Surgery, Asan Medical Center, University of Ulsan College of Medicine, 88 Olympic-Ro, 43-Gil, Songpa-Gu, Seoul, 05505 Republic of Korea; 2 Department of Gastroenterology, Ajou University Hospital, Ajou University School of Medicine, Suwon, Republic of Korea

**Keywords:** Hepatocellular carcinoma, Neoplasm metastasis, Parotid neoplasms, Positron emission tomography/computed tomography, Second primary neoplasms

## Abstract

**Objectives:**

We primarily aimed to evaluate whether parotid incidental lesion (PIL) in ^18^F-fluorodeoxyglucose positron emission tomography/computed tomography (^18^F-FDG PET/CT) for staging evaluation of patients with hepatocellular carcinoma (HCC) would represent a possibility of extrahepatic metastasis or second primary malignancy (SPM). Additionally, we explored the incidence of PIL in HCC patients and examined any associated risk factors.

**Methods:**

We retrospectively analyzed patients with HCC who underwent ^18^F-FDG PET/CT at our institution from 2010 to 2022. The pathological findings of PILs in HCC patients were investigated for confirmatory identification of the risk of HCC metastasis or SPM in parotid gland. Healthy controls received ^18^F-FDG PET/CT for health screening were also enrolled to compare the incidence of PILs with HCC patients. Various parameters associated with patient demographics and characteristics of HCC were analyzed to find the related factors of PILs.

**Results:**

A total of 17,674 patients with HCC and 2,090 healthy individuals who had undergone ^18^F-FDG PET/CT scans were enrolled in the analyses. Among the 54 HCC patients who underwent pathological confirmation for PILs, benign primary parotid tumor was most commonly observed (*n* = 43 [79.6%]); however, no malignant lesions were detected, including HCC metastasis. The incidence of PILs was higher in patients diagnosed with HCC compared with the control group (485 [2.7%] *vs.* 23 [1.1%], *p* = 0.002). Analysis for the risk factors for PILs revealed that patient age, sex, and positive viral markers were significantly associated with the incidence of PILs in patients with HCC (all *p* < 0.001).

**Conclusions:**

Our study demonstrates that PILs are more frequently identified in patients with HCC on ^18^F-FDG PET/CT. However, no malignant PIL, including extrahepatic metastasis of HCC, was identified. Therefore, the presence of PIL should not impede or delay the treatment process for patients with HCC. Additionally, we suggested that for future swift and straightforward differential diagnoses of PIL, the development of additional protocols within the PET/CT imaging could be beneficial.

## Background

^18^F-fluorodeoxyglucose (FDG) positron emission tomography/computed tomography (^18^F-FDG PET/CT) is a commonly employed imaging modality for staging various malignancies. PET/CT has excellent discriminative power to determine if the primary cancer has metastasized; however, sometimes a non-specific increase in FDG uptake is identified in the form of an incidentaloma, causing ambiguity in staging or requiring further diagnostic evaluation or consultation. In particular, FDG-avid benign tumors (e.g., Warthin tumor and oncocytoma) often occur in the parotid gland, wherein non-specific FDG uptake occasionally increases in the lymph nodes (LNs) or nearby parapharyngeal space. These spaces can therefore cause diagnostic difficulties for patients with cancer who undergo PET/CT [1]. According to the literature, parotid lesions, including incidentaloma, are found in 2.1% of patients with head and neck cancer during PET/CT scans, reporting 0.4% to 1.1% of cancers originating from outside the head and neck region is not uncommon [2–4].

Liver cancer is the sixth most common cancer in South Korea, and the major histopathology is hepatocellular carcinoma (HCC) [5]. At the time of diagnosis, HCC has often already reached an advanced stage, so accurately staging the cancer during initial evaluation is critical to determine the treatment modality and prognosis of the patients. Extrahepatic metastasis (EHM) is observed in approximately 13% of patients with HCC. In this case, the prognosis is extremely poor and a multidisciplinary approach is required to determine therapeutic strategies, it is therefore necessary to thoroughly check for metastasis before treatment [6, 7]. EHM primarily occurs in the lungs, bones, and intra-abdominal LNs or organs, but exactly when it occurs in the head and neck area remains undetermined [8]. Especially in HCC patients planning for hepatectomy or liver transplantation, it is crucial to assess the presence of EHM through PET/CT. In clinical practice, the discovery of parotid incidental lesion (PIL) in these patients is occasionally noted; however, there is a lack of prior research on whether these PILs represent important malignant conditions such as EHM of HCC or secondary primary malignancies (SPMs) in the salivary glands.

A remote lesion discovered incidentally during the staging step of a patient complicates the diagnostic process, delays treatment, and causes psychological anxiety for the patient. Therefore, investigating the frequency and histological examination results of the PIL observed on PET/CT in patients with HCC for whom no prior studies have been conducted will provide a guide for patient diagnosis and treatment decision-making. This study therefore aimed to analyze the frequency of PILs identified on PET/CT during HCC patient staging and investigate the pathological results of these lesions. In addition, we endeavored to analyze the characteristics of patients with HCC that affect the frequency of PILs.

## Materials & methods

### Patients and variables

We retrospectively analyzed the electronic medical records of patients with HCC who had undergone ^18^F-FDG PET/CT scans at our single tertiary referral hospital from 2010 to 2022. Patients with recurrent HCC or with a history of head and neck area malignancies, including primary parotid cancers, were excluded. The additional radiographs (including computed tomography, magnetic resonance image, or ultrasonography) and pathological evaluation of PILs through fine needle aspiration cytology or core needle biopsy in HCC patients were introduced to identify the risk of HCC metastasis or SPM in parotid gland. We also set a control group comprising patients who underwent ^18^F-FDG PET/CT scans for health screening during the same period to investigate the incidence of PIL compared with patients with HCC. To analyze the characteristics of HCC affecting the frequency of PILs, we inspected patient demographics (age, sex, smoking history, alcohol consumption amount) and HCC-associated parameters (tumor size as well as stage based on the Surveillance, Epidemiology, and End Results [SEER] classification, and alpha-fetoprotein [AFP] levels). Furthermore, we investigated whether the presence of hepatitis B virus (HBV) and hepatitis C virus (HCV) viral markers would affect the incidence of PIL, considering the previously reported association between head and neck squamous cell carcinoma and chronic HBV and HCV infections [9, 10]. This study was conducted with the approval of the Institutional Review Board of the hospital (IRB No. 2022–1684), and the requirement for patient consent was waived due to the retrospective nature of the analysis.

### Statistical analysis

Fisher's exact test or Student’s *t*-test was performed to compare the characteristics between patients with HCC and healthy controls with PIL. Logistic regression analysis was used to analyze the odds ratio (OR) of PIL in the patients with HCC. All statistical analyses were performed using IBM SPSS (ver. 22.0; IBM Corp, Armonk, NY), and statistical significance was defined as a two-sided *P*-value of less than 0.05.

## Results

### Incidence and pathologic findings of PIL

**Fig. 1:**
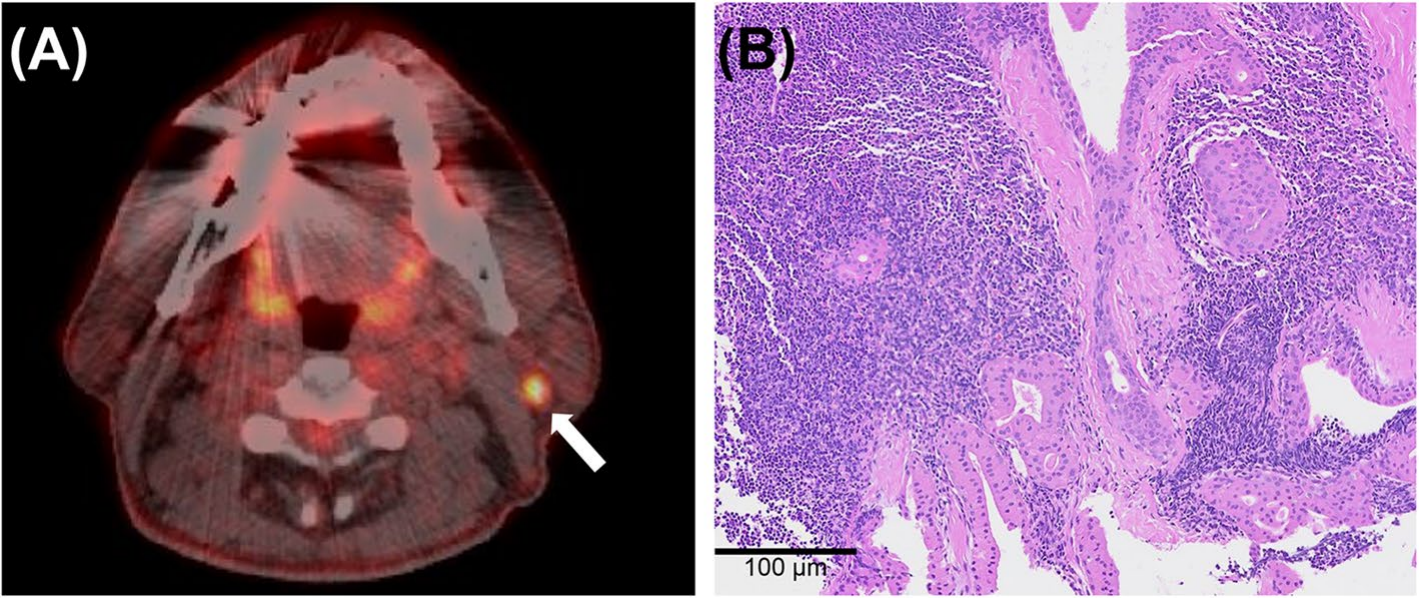
A representative patient case. **A** A representative case with a 50-year-old male undergoing staging workup for HCC having a focal hypermetabolic lesion in the left parotid gland (*white arrow*) with a maximum standardized uptake value of 5.5 on ^18^F-fluorodeoxyglucose positron emission tomography/computed tomography. **B** A core needle biopsy of the left parotid gland confirmed the presence of a Warthin tumor (hematoxylin & eosin stain, original magnification × 100)

Table 1 presents the demographics of all the patients. During the study period, 17,674 patients with HCC and 2,090 healthy individuals who underwent health checkups were enrolled. Among them, PET/CT scans revealed PIL in 485 (2.7%) patients with HCC (a representative case depicted in Fig. 1) and 23 (1.1%) in the control cohort, and the incidence of PIL was significantly higher in the patients with HCC (*p* < 0.001). We also performed statistical analysis by matching the two groups, given the differences in the age and sex distributions. The analyses revealed that 6,380 HCC group patients and 1,595 control group patients were matched at a 4:1 ratio. Even after matching, the occurrence of PIL in the HCC group (*n* = 118, 1.85%) was higher than in the control group (*n* = 15, 0.94%) (OR = 1.985, 95% CI: 1.157 − 3.406, *p* = 0.013, Table 1).

**Table 1:** Characteristics of subjects in each group

		**HCC group** (*n* = 17,674)		**Control group** (*n* = 2,090)		***P***	
Age (year), mean ± SD		58.7 ± 10.2		55.3 ± 10.3		< 0.001	
Sex, male/female		14,315 (81) / 3359 (19)		920 (44) / 1170 (56)		< 0.001	
Smoking history never ever or current unknown		946 (5.4) 3542 (20) 13,186 (74.6)		686 (32.8) 226 (10.8) 1178 (56.4)		< 0.001	
Alcohol intake never social or heavy unknown		3131 (17.7) 6599 (37.3) 7944 (45)		221 (10.6) 16 (0.8) 1853 (88.7)		< 0.001	
HBsAg negative positive unknown		10,606 (60) 1886 (10.7) 5155 (29.2)		N/A		N/A	
Anti-HCV antibody negative positive unknown		12,253 (64.4) 239 (13.5) 5155 (29.2)		N/A		N/A	
AFP (ng/ml), mean ± SD		10,121.1 ± 91,585.4		N/A		N/A	
SEER stage DM ( −) DM ( +) unknown		15,580 (88.2) 1007 (5.7) 1086 (6.2)		N/A		N/A	
PIL		485 (2.7)		23 (1.1)		< 0.001	
	**HCC group (*n* = 6,380)**		**Control group (*n* = 1,595)**		**OR (95% CI)**		***P***
PIL^a^	118 (1.85)		15 (0.94)		1.985 (1.157 − 3.406)		0.013

Data expressed as number (%)

*AFP* alpha-fetoprotein, *CI* confidence interval, *DM* distant metastasis, *HBsAg* hepatitis B surface antigen, *HCC* hepatocellular carcinoma, *HCV* hepatitis C virus, *N/A* not assessable, *OR* odds ratio, *SD* standard deviation, *SEER* surveillance, epidemiology, and end result, *PIL* parotid incidental lesion

^a^After matching at a 4:1 ratio to adjust for differences in age and sex distribution between the two groups

**Fig. 2:**
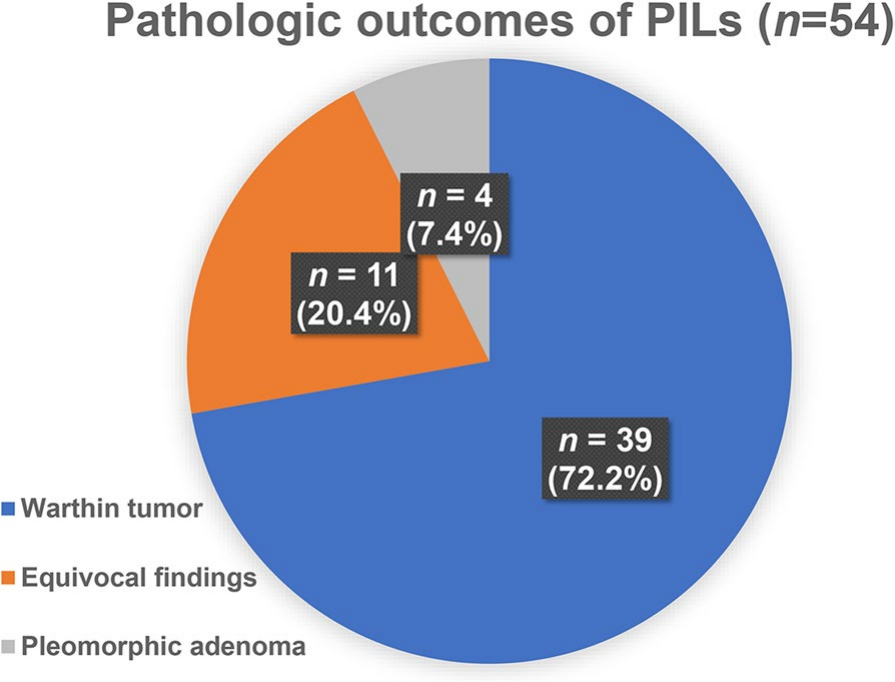
The pathological outcomes of parotid incidental lesions (PIL, *n* = 54)

Out of 485 cases of PIL, in instances where the PET/CT interpreting physician recommended further evaluation for differential diagnosis, additional radiographs and tissue examinations were conducted. However, actual histopathological confirmations were performed in only 54 (11.1%) cases.. The most common pathologic finding was Warthin tumor (*n* = 39, 73.6%), followed by equivocal results (e.g., lymphoid tissue, abscess, necrotic tissue, reactive hyperplasia, or acellular tissue [*n* = 11, 8.8%]), and pleomorphic adenoma (*n* = 4, 7.6%); however, no malignant lesions were observed, including EHM of HCC (Fig. 2).

### Risk factors for PIL in HCC patients

We performed another comparison within patients with HCC to investigate the risk factors associated with the presence of PIL. Risk factors included older age, male sex, and HBsAg and anti-HCV positivity (all *p* < 0.001). In the logistic regression analysis, age (OR = 1.028, 95% CI: 1.019 − 1.037, *p* < 0.001), male sex (OR = 3.082, 95% CI: 2.181 − 4.357, *p* < 0.001), HBsAg positivity (OR = 6.891, 95% CI: 5.447–8.717, *p* < 0.001), and anti-HCV positivity (OR = 3.776, 95% CI: 2.328 − 6.125, *p* < 0.001) significantly increased the risk of PILs. However, initial tumor size, AFP level, and SEER stage did not yield any statistically significant association with PIL presence (Table 2).

**Table 2:** Univariate and logistic regression analyses on parameters affecting the prevalence of parotid incidental lesion

**Variables**	**PIL ( +)** (*n* = 485)	**PIL ( −)** (*n* = 12,199)	***P***	**OR (95% CI)**	***P***
Age (years), mean ± SD	61.4 ± 9.7	58.6 ± 10.3	< 0.001	1.028 (1.019–1.037)	< 0.001
Sex, male/female	450 (92.8) / 35 (7.2)	13,865 (80.7) / 3324 (19.3)	< 0.001	3.082 (2.181–4.357)	< 0.001
HBsAg, positive/negative	156 (53.2) /137 (46.8)	1730 (14.2) / 10,469 (85.8)	< 0.001	6.891 (5.447 − 8.717)	< 0.001
Anti-HCV, positive/negative	19 (6.5) / 274 (93.5)	220 (1.8) / 11,979 (98.2)	< 0.001	3.776 (2.328–6.125)	< 0.001
AFP (µg/mL), mean ± SD	8.772 ± 84.92	7.455 ± 73.98	0.764	1 (1 − 1)	1
HCC size (cm), mean ± SD	4.5 ± 3.5	4.6 ± 8.9	0.932	0.999 (0.983 − 1.015)	0.932
SEER stage, DM( −)/DM( +)	290 (93.5) / 16 (5.5)	11,518 (94.4) / 599 (4.9)	0.656	1.173 (0.732 − 1.879)	0.507

Data expressed as number (%)

Blood tests about HBsAg, Anti-HCV, and AFP were performed within 3 months before PET/CT

*AFP* alpha-fetoprotein, *DM* distant metastasis, *HBsAg* hepatitis B surface antigen, *HCC* hepatocellular carcinoma, *HCV* hepatitis C virus, *PIL* parotid incidental lesion, *SEER* surveillance epidemiology and end result

## Discussion

In patients with HCC, tumor stage is used to predict the clinical course and determine the treatment method. EHM of HCC is not uncommon, and the probability of identifying EHM is higher in patients with advanced intrahepatic HCC. The prognosis of these patients is poor, and a systemic agent such as sorafenib is the only therapeutic option [11]. To accurately determine the HCC stage, a test for EHM is required, for which ^18^F-FDG PET/CT is an established tool [12, 13]. Moreover, upstaging reportedly occurred in 5.9% patients with HCC, among which EHM was identified in 1.4% when PET/CT was performed before the initial treatment, consequently leading to the changed treatment policy in 9.9% of those patients [14, 15]. Therefore, PET/CT should be actively considered in patients with HCC, especially when planning surgical treatment for curative purposes, such as hepatectomy or liver transplantation.

According to a recent systematic review, the overall prevalence of PIL in various primary tumor staging examinations using ^18^F-FDG PET/CT was 0.74%, with a significantly higher frequency observed in patients with lung cancer [16]. In our study, PIL was detected in 2.7% of patients with HCC, showing a higher prevalence compared with other non-head and neck primary cancers, and a higher likelihood of detection in patients with head and neck cancer (2.1%) [3]. The etiology of HCC primarily includes hepatitis virus infection, as well as factors such as male sex, old age, smoking, and alcohol consumption, which are also risk factors for primary parotid tumors [17]. Therefore, it is presumed that the higher frequency of such risk factors in the HCC group compared to the control group, might have contributed to the difference in PIL incidence. However, even after adjusting for the aforementioned common risk factors, the higher frequency of PIL in the HCC group compared to the control group suggests that HCC itself may increase the incidence of PIL.

According to the aforementioned systematic review by Thompson and colleagues, the majority of tumors found in cases wherein histological confirmation was available were benign, such as Warthin tumor (cystadenoma). However, malignant lesions were identified in approximately 30% of cases, including metastatic LNs and primary parotid gland malignancies [16]. In contrast, among the 54 patients in our study who underwent histological confirmation for PIL, no malignant lesions were found. Case reports of HCC metastasis to the head and neck region are rarely confirmed justly in the literature. While metastasis to the oral or pharyngeal mucosa is occasionally reported, there was only one case of metastasis to the parotid gland reported [18–22]. Collectively, while the incidence of PIL in patients with HCC on PET/CT is relatively higher, the likelihood of detecting malignant lesions, including EHM of HCC, appears to be extremely low.

In our study, we confirmed that PIL was highly likely to be identified if the patient with HCC had risk factors such as male sex, old age, positive HBsAg, or anti-HCV. An interesting observation is that the frequency of PIL was higher in HCC cases with positive markers for hepatitis viruses. Hepatitis virus infection is a proven risk factor for the development of another extrahepatic primary malignancy [23, 24]. This is attributed to the presence of factors such as hepatitis B virus X protein, which can lead to genetic instability and an immunosuppressive condition, thereby increasing the risk of developing malignancies in other organs [25]. Our study was initiated based on previous literature suggesting that hepatitis virus infection also increases the risk of developing head and neck cancer [9, 10]. We therefore wondered whether the presence of PIL in hepatitis virus-positive patients with HCC could lead to a higher risk not only for EHM but also for the development of secondary primary salivary gland cancer. However, in our study, we did not observe any cases of EHM or primary salivary gland cancer in the PIL confirmed through tissue examination. Most of the non-malignant lesions were salivary gland-origin benign tumors, with Warthin tumors being the most commonly observed among them. Warthin tumor is associated with predisposing factors such as male sex, old age, and heavy smoking. Recently, there have been reports linking it to infections of human papillomavirus or Epstein–Barr virus, as well as associations with chromosomal instability [26, 27]. Considering these factors, it is also plausible to speculate about a potential association between Warthin tumor development and hepatitis virus infection to explain the phenomenon of an increased frequency of PIL and benign salivary gland tumors in HCC patients with positive hepatitis virus markers. Despite the insights gained from our study, further research is warranted to gain a comprehensive understanding of the underlying mechanisms contributing to the higher incidence of PIL in HCC patients.

The main limitation of this study is its retrospective nature. The inability to ascertain the status of patients who did not undergo histological examinations is a crucial limitation of our study. Given the retrospective design of this study, when the PET/CT interpreting physician did not recommend additional tests for differential diagnosis among the 485 cases of PIL, most patients were not referred separately to the otolaryngology department or did not undergo further testing for PIL. This made it challenging to assess their condition. And the comparison of incidence rates and identification of risk factors for PIL were conducted in different populations, which subsequently led to the limited data set and comparisons. Additionally, the use of ^18^F-FDG PET/CT scans in patients with HCC is somewhat limited to those scheduled for surgical treatment owing to the insurance system and liver cancer treatment guidelines in our country. Another limitation of our study would be lack of analysis the relationship between quantitative FDG uptake of variety benign parotid tumors. However, our study represents the first report confirming the relatively higher incidence and totally benign pathologic results of PIL in patients with HCC. Our study can offer valuable guidance on the need for additional examinations and otolaryngology consultations, especially in patients with HCC who are candidates for urgent hepatectomy or liver transplantation.

## Conclusion

We observed that ^18^F-FDG PET/CT scans identified PIL more frequently in patients with HCC compared with healthy controls, with a prevalence of 2.7% and 1.1%, respectively. Several factors influence the incidence of PIL in patients with HCC, including older age, male sex, positive HBsAg status, and positive anti-HCV status. Notably, all of PIL detected and tissue-confirmed in patients with HCC was benign, given that no malignant lesions, including EHM, were identified. Therefore, the presence of PIL should not impede or delay the treatment process for patients with HCC. Additionally, we suggested that for future swift and straightforward differential diagnoses of PIL, the development of additional protocols within the PET/CT imaging could be beneficial.

## Data Availability

The datasets generated and/or analysed during the current study are not publicly available due to the institutional policy regarding the patients’ information but are available from the corresponding author on reasonable request.
